# *In Vitro* Antiviral Activity of Circular Triple Helix Forming Oligonucleotide RNA towards Feline Infectious Peritonitis Virus Replication

**DOI:** 10.1155/2014/654712

**Published:** 2014-02-20

**Authors:** Oi Kuan Choong, Parvaneh Mehrbod, Bimo Ario Tejo, Abdul Rahman Omar

**Affiliations:** ^1^Institute of Bioscience, Universiti Putra Malaysia, 43400 Serdang, Selangor, Malaysia; ^2^Faculty of Veterinary Medicine, Universiti Putra Malaysia, 43400 Serdang, Selangor, Malaysia; ^3^Faculty of Science, Universiti Putra Malaysia, 43400 Serdang, Selangor, Malaysia

## Abstract

Feline Infectious Peritonitis (FIP) is a severe fatal immune-augmented disease in cat population. It is caused by FIP virus (FIPV), a virulent mutant strain of Feline Enteric Coronavirus (FECV). Current treatments and prophylactics are not effective. The *in vitro* antiviral properties of five circular Triple-Helix Forming Oligonucleotide (TFO) RNAs (TFO1 to TFO5), which target the different regions of virulent feline coronavirus (FCoV) strain FIPV WSU 79-1146 genome, were tested in FIPV-infected Crandell-Rees Feline Kidney (CRFK) cells. RT-qPCR results showed that the circular TFO RNAs, except TFO2, inhibit FIPV replication, where the viral genome copy numbers decreased significantly by 5-fold log_10_ from 10^14^ in the virus-inoculated cells to 10^9^ in the circular TFO RNAs-transfected cells. Furthermore, the binding of the circular TFO RNA with the targeted viral genome segment was also confirmed using electrophoretic mobility shift assay. The strength of binding kinetics between the TFO RNAs and their target regions was demonstrated by NanoITC assay. In conclusion, the circular TFOs have the potential to be further developed as antiviral agents against FIPV infection.

## 1. Introduction

Feline Infectious Peritonitis Virus (FIPV) is an enveloped virus with a nonsegmented, positive sense, single-stranded RNA genome. FIPV is grouped as feline coronavirus (FCoV), under the family Coronaviridae. FCoV is divided into two biotypes, namely, Feline Enteric Coronavirus (FECV), a ubiquitous enteric biotype of FCoV, and FIPV, a virulent biotype of FCoV [1]. The relationship between these two biotypes still remains unclear. Two hypotheses have been proposed, (i) internal mutation theory and (ii) circulating high virulent-low virulent theory. Internal mutation theory stated that the development of FIP is due to the exposure of cat to variants of FCoV which have been mutated by gaining the ability to replicate within the macrophages [2], while the circulating high virulent-low virulent theory explains the existence of both distinctive pathogenic and benign lineages of viruses within the cat population [3].

Study has shown that about 40–80% of cats are detected with FECV shedding in their faeces [4]. About 12% of these FECV-positive cats have developed immune-mediated fatal FIP disease [4]. The prevalence of FIP among felines is due to continual cycles of infection and reinfection of FECV and indiscernible clinical symptoms of infected cats with FECV at an early stage before the progressive development of FIPV.

Vaccination against FIPV with an attenuated, temperature-sensitive strain of type II FIPV induces low antibody titre in kittens that have not been exposed to FCoV. However, there is considerable controversy on the safety and efficacy of this vaccine, since the vaccine contains type 2 strain, whereas type 1 viruses are more prevalent in the field [4]. In addition, antibodies against FIPV do not protect infected cats but enhance the infection of monocytes and macrophages via a mechanism known as Antibody-Dependent Enhancement [1]. Besides vaccines, several antiviral drugs such as ribavirin, interferons, and immunosuppressive drugs have been used as treatments for FIPV-infected cats, mainly to suppress the inflammatory and detrimental immune response [5–8]. However, those treatments were ineffective. Hence, there is still significant unmet medical need to develop effective treatments and prophylactics for FIPV infection.

Triple Helix Forming Oligonucleotide (TFO) is defined as homopyrimidine oligonucleotides, which can form a sequence-specific triple helix by Hoogsteen bonds to the major groove of a complementary homopyrimidine-homopurine stretch in duplex DNA [9]. Furthermore, double helical RNA or DNA-RNA hybrids can be targeted as a template for triple helix formation, once the strand composition on the stabilities of triple helical complexes is determined [10]. Hence, TFO has been used to impede gene expressions by transcription inhibition of viral genes or oncogenes [11–16]. The main purpose of this study is to develop and evaluate the *in vitro* antiviral properties of circular TFO RNAs against FIPV replication.

## 2. Materials and Methods

### 2.1. Cell and Virus

Feline Infectious Peritonitis Virus (FIPV) serotype II strain WSU 79–1146 (ATCC no. VR-1777) was grown in CRFK cells. A serial 10-fold dilution of FIPV was prepared from the working stock. Confluent 96-well plate was inoculated with 100 *μ*L of each virus dilution/well. The plate was incubated in a humidified incubator at 37°C, 5% CO_2_. Cytopathic effects (CPE) development was observed. The results were recorded after 72 hours and the virus tissue culture infective dose 50 (TCID_50_) was calculated using Reed and Muench's method [17].

### 2.2. Preparation of Circular Triple Helix Forming Oligonucleotide RNA

The Triple Helix Forming Oligonucleotides (TFOs) were designed based on the genome sequence of FIPV serotype II strain WSU 79–1146 (Accession no: AY994055) [18]. TFOs, which specifically target the different regions of the FIPV genome, and one unrelated TFO were constructed (Table 1). The specificity of the TFOs was identified using BLAST search in the NCBI database. The designed linear TFOs were synthesized by Dharmacon Research (USA), whereby the 5′ and 3′ ends of the linear TFOs were modified with phosphate (PO_4_) group and hydroxide (OH) group, respectively. These modifications were necessary for the circularization of linear TFO. The process of circularization, using the T4 RNA ligase 1 (ssRNA ligase) (New England Biolabs Inc., England), was carried out according to the manufacturer's protocol. After ligation, the circular TFO RNAs were recovered by ethanol precipitation and the purity of the circular TFO RNAs was measured using spectrophotometer.

### 2.3. Denaturing Polyacrylamide Gel Electrophoresis

Denaturing of urea polyacrylamide gel electrophoresis was performed as described before [19] with modification. Briefly, 20% of denatured urea polyacrylamide gel was prepared and polymerized for 30 minutes. Then, the gel was prerun at 20 to 40 V for 45 minutes. Five *μ*L of TFO RNA mixed with 5 *μ*L of urea loading buffer was heated at 92°C for 2 minutes and immediately chilled on ice. It was run on the gel at 200 V for 45 minutes. Finally, the gel was stained with ethidium bromide (Sigma, USA) and viewed with a Bio-Rad Gel Doc XR system (CA, USA).

### 2.4. Electrophoretic Mobility Shift Assay (EMSA)

The target regions of the FIPV genome were synthesized by Dharmacon Research (USA) (Table 1). Each TFO RNA was mixed with the target region in 1X binding buffer containing 25 mM Tris-HCl, 6 mM MgCl_2_, and 10 mMNaCl in a final volume of 10 *μ*L and subsequently incubated at 37°C for 2 hours. The sample was run on 15% native polyacrylamide gel at 80 V, in cool condition. The stained gel was viewed by a Bio-Rad Gel Doc XR system.

### 2.5. Interaction between Circular TFO RNAs and the Target Regions

The binding strength was measured using a nano Isothermal Titration Calorimeter (ITC) (TA instruments, Newcastle, UK). The RNA sample mixtures, consisting of circular TFOs (0.0002 mM), were incubated with their respective synthetic target regions (0.015 mM) using 1X binding buffer as the diluent. The experiment was run at 37°C with 2 *μ*L/injection, for a total of 25 injections. Data was collected every 250 seconds and analyzed using the NanoAnalyze software v2.3.6 provided by the manufacturer.

### 2.6. *In Vitro* Antiviral Effect of TFOs towards FIPV

This experiment was conducted in CRFK cells, where 3 × 10^4^ cell/well was seeded in 96-well plate to reach 80% confluency 24 hours prior to transfection. One hundred nM of TFO RNAs was separately transfected into the CRFK cells using a HiPerFect Transfection Reagent (Qiagen, Germany), as per the manufacturer's protocol. The plate was incubated at 37°C with 5% CO_2_ for 6 hours. Then, the cultures were infected with 100TCID_50_ of FIPV serotype II strain WSU 79–1146 for 1 hour at 37°C (100 *μ*L/well). Finally, the viral inoculum was replaced by fresh maintenance media (MEM containing 1% FBS and 1% pen/strep). Virus-infected and uninfected cells were maintained as positive and negative controls, respectively. The morphology of the cultures was recorded 72 hours after infection and samples were harvested at this time point and stored at −80°C prior to RNA extraction.

### 2.7. Dose-Response of Circular TFO RNA in FIPV Replication Inhibition

Different concentrations of circular TFO1 RNA (25 nM, 50 nM, 100 nM, and 500 nM) were transfected into CRFK cells. The plate was incubated for 6 hours followed by virus inoculation for 1 hour at 37°C with 5% CO2. The cells were processed as described above.

### 2.8. *In Vitro* Specificity Study of Circular TFO RNAs towards Influenza A Virus

Madin-Darby Canine Kidney (MDCK) cell (ATCC no. CCL-34), at a concentration of 4 × 10^4^ cell/well, was seeded in 96-well plate to reach 80% confluency 24 hours prior to transfection. Transfection was performed the same as before. One hundred nM of circular TFO RNA was transfected into MDCK cells. Following 6 hours incubation, the solution was discarded and 100TCID_50_ of influenza A virus subtype H1N1 New Jersey 8/76 (ATCC VR-897) was inoculated into the plate for 1 hour at 37°C. The viral inoculums were replaced by Dulbecco's Modified Eagle's Medium (DMEM) (Mediatech Cellgro Company; Northbrook, Illinois, USA), containing Trypsin-(Tosylamide-2-Phenylethyl Chloromethyl Ketone) TPCK (Sigma, USA). Samples were harvested after 48 hours and stored at −80°C prior to RNA extraction.

### 2.9. Reverse Transcriptase Quantitative Real-Time PCR

The viral RNA was extracted from 100 *μ*L of the harvested cell culture using a QIAamp viral RNA extraction kit (Qiagen, Germany), as per the manufacturer's protocol. Virus-infected and uninfected cells were maintained as positive and negative controls, respectively. The primers for amplification of FIPV and H1N1 (Table 2) were based on the established primers previously designed [20, 21], respectively. The reverse transcriptase quantitative real-time PCR (RT-qPCR) was performed using a Bio-Rad CFX96 real-time system (BioRad, USA). The reaction was amplified in a final volume of 25 *μ*L using a SensiMix SYBR No-ROX One-Step Kit (Bioline, UK), which consisted of 12.5 *μ*L 2X SensiMix SYBR No-Rox One-Step reaction buffer, 10 *μ*M forward and reverse primers, 10 units RiboSafe RNase inhibitor, and 5 *μ*L template RNA. Absolute quantification approach was used to quantify qPCR results where a standard curve of a serial dilution of virus was plotted before the quantification. Amount of the virus in the samples was quantified based on this standard curve.

### 2.10. Statistical Analysis

Data statistical analysis was performed using SPSS 18.0. Data were represented as mean ± SE of three independent tests. One-way ANOVA, Tukey post hoc test was used to analyze the significant level among the data. *P* ≤ 0.05 was considered significant.

## 3. Results 

### 3.1. Design of Anti-FIPV Circular TFO RNAs

Circular TFO RNAs targeting FIPV genome were designed according to the triple helix formation method described by Vo and colleagues [22] with modification. Three different regions of the FIPV genome, which play important roles in viral replication, were selected as the target binding sites for the triplex formation. The target regions were 5′ untranslated region (5′ UTR), Open Reading Frames (ORFs) 1a and 1b, and 3′ untranslated region (3′ UTR) (Table 1). The TFOs were designed in duplex, as they can bind with the single stranded target region and reshape into triplex. Both ends of the duplex TFOs were ligated with a linker sequence or clamps (C-C) to construct circular TFO RNA.

### 3.2. Determination of Circular TFO RNA

**Figure 1:**
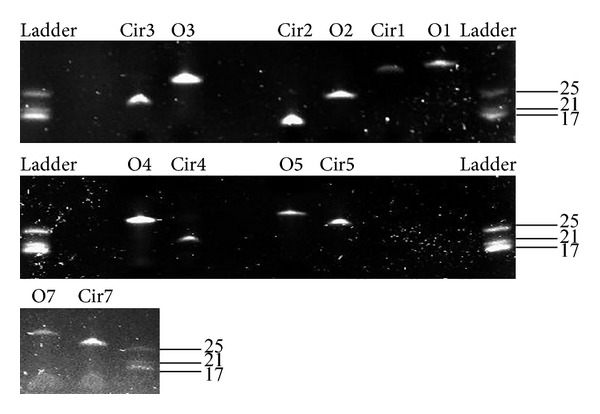
Denaturing PAGE to determine circular TFO RNA. Denaturing PAGE analysis confirmed that linear TFOs were successfully circularized, whereas circular TFOs migrated faster than linear TFOs; however, only slight variation was detected for some of the TFOs. O1 to O7 represent linear TFO1 to TFO7, while Cir1 to Cir7 represent circular TFO1 to TFO7.

Denaturing PAGE assay was carried out after the ligation process to determine the formation of the circular TFO. As shown in Figure 1, the circular TFO RNAs migrated faster than the linear TFO RNAs, when subjected to 20% denaturing PAGE.

### 3.3. The Binding Ability of Circular TFO RNA towards Its Target Region

**Figure 2:**
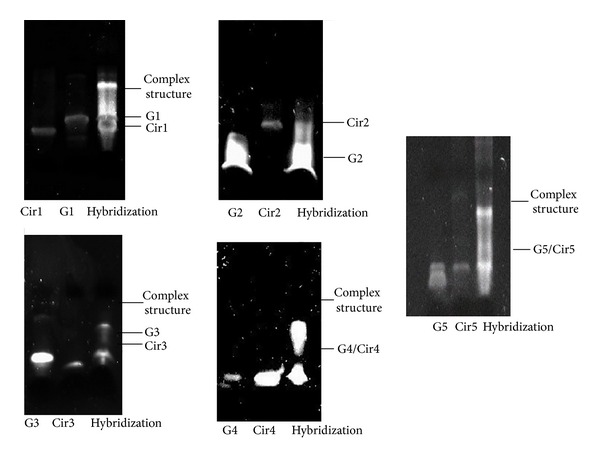
EMSA analysis. EMSA analysis illustrated the binding of circular TFO1, 3, 4, and 5 to the target regions as evidenced by upward band shift. Binding of each circular TFO except circular TFO2 to its respective target forms a complex that migrates slower than unbound TFO. G1 to G5 represent the target region for circular TFO1 to TFO5 and Cir1 to Cir5 represent the circular TFO1 to TFO5, respectively.

The binding ability was determined using Electrophoretic Mobility Shift Assay (EMSA) [23]. The appearance of the slow mobility band indicates the successful hybridization of circular TFO RNA with its target region. The binding ability of different TFO RNAs (TFO1 to TFO5) against their target regions was determined by EMSA (Figure 2). TFO1, TFO3, TFO4, and TFO5 showed slow mobility band, while TFO2 showed the lack of an upward shifted band. This indicates the possession of triplex binding ability for all circular TFO RNAs, except TFO2.

### 3.4. Nano Isothermal Titration Calorimeter Study of Circular TFO RNA

Study on the interaction and hybridization of TFO towards its target region is crucial, since the stronger the binding is, the more stable the triplex structure forms. As shown in supplementary Figure 1 (in Supplementary Materials available online at http://dx.doi.org/10.1155/2014/654712), all circular TFO RNAs (except TFO2) showed high levels of association constant (*K*_*a*_). Consequently, all circular TFO RNAs (except TFO2) recorded a low dissociation constant (*K*_*d*_) value, in a reverse relation with the *K*_*a*_ value. TFO1 showed the lowest *K*_*d*_ value (Table 3).

### 3.5. Antiviral Effect of Circular TFO RNAs against FIPV

**Figure 3:**
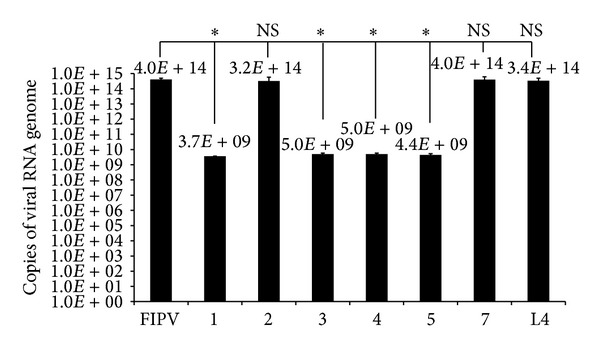
RT-qPCR analysis. RT-qPCR assay confirmed the inhibition of FIPV replication by circular TFO RNAs, except TFO2. Data are averages of 3 independent tests (mean ± SE). 1 to 5 represent circular TFO1 to TFO5, 7 represents unrelated circular TFO7, and L4 represented the linear TFO4. *Significantly different from value obtained for TFOs treatments compared to FIPV infected cells (*P* ≤ 0.05). NS: not significant.

The antiviral effect of circular TFO RNAs was investigated by RT-qPCR assay at 72 hours after transfection. The results showed viral RNA genome copy numbers of 3.65 × 10^9^, 3.22 × 10^14^, 5.04 × 10^9^, 5.01 × 10^9^, 4.41 × 10^9^, and 3.96 × 10^14^ in cells treated with TFO1, TFO2, TFO3, TFO4, TFO5, and TFO7, respectively. The data analyzed by one-way ANOVA, Tukey post hoc test showed significant high viral RNA genome copy number of 4.03 × 10^14^ for virus inoculated cells as compared to circular TFO1, TFO3, TFO4, and TFO5 treatments (*P* ≤ 0.05). The viral RNA copies of circular TFO2, linear TFO3 and TFO4, and unrelated circular TFO7 RNAs transfected cells also showed high viral RNA copy numbers which did not show significant differences to the infected cells (*P* ≥ 0.05) (Figure 3). The morphological changes of the cells were also captured 72 hours after transfection. The cells transfected with circular TFO1, TFO3, TFO4, and TFO5 appeared to be in good condition following virus inoculation, while the cells transfected with circular TFO2 and linear TFO3 and TFO4 showed visible cytopathic effect (CPE), the same as virus inoculated cells (supplementary Figure 2). Furthermore, cells transfected with TFO only remain viable indicating that TFO treatment is generally not toxic to the cells. Hence, these results illustrated the capacity of circular TFO RNAs (except TFO2) to inhibit FIPV replication.

### 3.6. Effect of Circular TFO RNA in Different Concentrations on FIPV Replication

**Figure 4:**
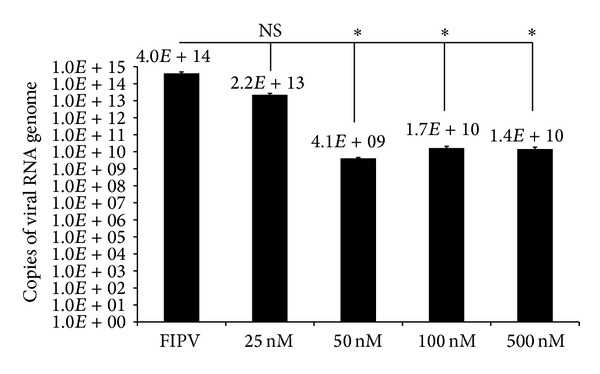
TFO1 dose-response study for inhibiting FIPV replication. The concentrations of 50 nM and higher showed significant antiviral effects. 50 nM of circular TFO1 RNA was able to reduce viral copy number by 5-fold log_10_ from 10^14^ to 10^9^, while 100 and 500 nM showed 4-fold reduction. Data are averages of 3 independent tests (mean ± SE). *Significantly different from FIPV-infected group.

Circular TFO1 was used to examine the dose-response relationship as a representative to other TFOs. The experimental conditions were identical to that of the previous experiment, except for TFO1 concentrations of 25 nM, 50 nM, 100 nM, and 500 nM. There was no significant reduction in viral RNA genome copies using the concentration of 25 nM TFO1. The other concentrations caused significant reductions in copy numbers as compared to the virus-infected cells. However, no significant difference was detected in copy numbers from all of these concentrations (Figure 4).

### 3.7. Antiviral Effect of Circular TFO RNAs on Unrelated Virus

The specificity of the TFO towards FIPV was tested, using TFO1 and TFO5, as the proper representatives of TFOs, on influenza A virus H1N1 New Jersey 8/76. The analyzed data using one-way ANOVA, Tukey post hoc test did not show significant reductions in the copies of viral RNA for both TFOs compared to the influenza virus inoculated cells (*P* ≥ 0.05) (supplementary Figure 3).

## 4. Discussion and Conclusion

Feline Infectious Peritonitis is a fatal disease in cat population. Current treatments and vaccines are ineffective to control the disease. The 5′ and 3′ UTRs of the virus mRNA are highly conserved sequences and play an important role in the replication process [24]. Meanwhile, the ORF1a/1b of FIPV are translated into polyproteins that are cleaved into nonstructural proteins which assemble into replication-transcription complexes together with other viral proteins [24]. Hence, the development of molecular therapy targeting these critical regions may provide the possibility to inhibit FIPV replication.

Development of antiviral therapies against FIPV using siRNA [25] and viral protease inhibitors [26] has been tested as potential new treatments against FIPV infection. In this study, circular Triple Helix Forming Oligonucleotide (TFO) RNAs, specifically targeting the short regions of viral genome for triplex formation, were designed and evaluated. TFO1 and TFO2 targeted the 5′ and 3′ UTRs of the viral genome, respectively. TFO3 to TFO5 targeted different regions of the ORF1a/1b on FIPV genome. Prior to *in vitro* antiviral study, the ligated circular TFOs were evaluated using PAGE analysis. All of the circularised TFO showed faster migration pattern compared to the linear TFO; however, only slight variation was detected for some of the TFO (Figure 1). The reason for this is not clear but probably due to the differences in length and the tertiary structures of the TFOs leading to differences in the migration rate. EMSA was used to show the binding capability of each circular TFO towards the target region in the FIPV genome except for TFO2 which showed lack of formation of complex structure upon hybridization (Figure 2). The EMSA result also concurred with the antiviral study, where all circular TFOs (except TFO2) were able to demonstrate a significant reduction in the viral RNA genome copy numbers by 5-fold log_10_ from 10^14^ in virus inoculated cells to 10^9^ in TFO-transfected cells (Figure 3). However, no antiviral properties were detected from the linear TFOs and unrelated circular TFO7 RNA, confirming that the antiviral activity is associated with specific binding of circular TFOs towards targeted regions.

Furthermore, the binding of the circular TFO to the target region was confirmed by nanoITC analysis; where the low *K*_*d*_ value and high stability allowed TFOs to compete effectively with the target regions for inhibiting transcription in cell-free systems. Since, TFO1 shows the lowest *K*_*d*_ value (Table 3), the antiviral properties of this TFO were evaluated in dose-response study. As shown in Figure 4, 50 and 100 nM of TFO1 showed similar antiviral effects indicating the potential therapeutic application of TFO1 on FIPV replication. However, increasing the concentration of TFO1 to 500 nm failed to reduce the viral load further probably due to inefficiency of the transfection reagent to transfect the TFO into the cells. In addition, the virus has fast replication rate upon *in vitro* infection, where previous study on the growth of FIPV in CRFK cells showed that by 2 hours approximately 67% of FIPV 79-1146 were internalized by CRFK cells by endocytosis increasing to more than 70% at 3 hours [27, 28]. The above finding probably also explained the reason why no antiviral effect was detected when the transfection of the TFO was performed on virus-infected cells (data not shown).

The antiviral properties, as demonstrated by the circular TFOs, were probably associated with the binding of the TFO to the target region, based on both the Watson-Crick and Hoogsteen hydrogen bonds, which enhance the stability in terms of enthalpy, which is brought about by joining together two out of three strands of the triple helix in the proper orientation [29]. Therefore, the triplex formation is tightly bonded and not easy to detach. Furthermore, the circular TFOs were designed in such way that the presence of hydrogen bonding donors and acceptors in the purines is able to form two hydrogen bonds, while the pyrimidine bases can only form one additional hydrogen bond with incoming third bases [30]. However, there are various factors that may limit the activity of TFOs in cells like intracellular degradation of the TFO and limited accessibility of the TFO to the target sites which can prevent triplex formation [31]. These findings may also explain the inability of the designed TFO1 to inhibit further virus replication in dose-response study (Figure 4).

Various molecular-based therapies against infectious diseases and cancer have been developed and tested. However, only the siRNA-based therapy has been studied extensively as a novel antiviral and anticancer therapy [32, 33]. Recently, McDonagh et al. [25] developed siRNA with antiviral activity against the FIPV 79-1146, where the designed siRNA was able to reduce the copy number of viral genome compared with virus-infected cells. The potential therapeutic application of TFOs, such as linear TFO conjugated with psoralen to inhibit the transcription of human immunodeficiency provirus [13] and TFO to inhibit the transcription of *α*1(I) collagen in rat fibroblasts [14], has also been reported. In addition, short TFO conjugated with daunomycin targeting the promoter region of oncogene has been designed and evaluated on human cancer cells [31]. These studies indicated the flexibility of using TFO-based oligonucleotides as a potential molecular-based therapy. In this study, we demonstrated short circular TFO RNAs between 28 and 34 mers (Table 1), which are able to inhibit FIPV replication by binding to specific target regions of the FIPV genome.

All designed circular TFOs (except TFO2) showed significant inhibitory effects against FIPV replication. The TFOs that formed triplex structures showed antiviral effects towards FIPV replication. The reason why TFO2 failed to show any interaction with the target region or antiviral activity is probably due to the length of TFO2 (i.e., 24 mers), which might be insufficient to a triplex formation upon hybridization (Figure 2), be effective enough to suppress viral RNA transcription, and eventually inhibit virus replication. Nevertheless, the inability of TFO2 to show antiviral effect due to failure in the formation of functional tertiary structure of the triplex formation cannot be ruled out. *In vitro *antiviral study which showed no antiviral property for unrelated TFO (TFO7) and also inability of circular TFO1 and TFO5 to inhibit influenza A virus H1N1 infected cells confirms the specificity of the TFOs' activity.

In conclusion, the circular TFO RNA has the potential to be developed as a therapy against FIPV in cats. However, further studies on TFO specificity, actual mechanism of circular TFO RNA in the transcription alteration consequence of inhibiting the viral transcription process, and *in vivo* animal studies are important for this approach to work as a therapy in the future.

## Supplementary Material

Supplementary Figure 1. NanoITC analysis. NanoITC analysis measures the binding affinity of circular TFO to its target region. Dissociation constant (Kd) is equal to 1/association constant (Ka). Supplementary Figure 2. Morphological changes of CRFK cells following TFO RNAs treatment. FIPV-infected cells exposed to TFOs which did not show cytopathic effect (CPE) indicated the inhibitory effects of the circular TFO against FIPV replication. Arrows show the CPE. 10X magnification. Supplementary Figure 3. In vitro antiviral activity of circular TFO. In vitro antiviral activity of TFO1 (A) and TFO5 (B) towards influenza A virus H1N1 replication showed no significant reduction in viral RNA genome copy numbers between the TFO RNA transfected cells and untransfected cells (*p* ≥ 0.05). Data are averages of 3 independent tests (mean ± SE). NS: not significant.

## Figures and Tables

**Table 1:** Sequences of FIPV-specific circular TFOs.

TFOs	TFO sequences	Target sequence*	Target gene and position
TFO1	GAGAAGAAAAGGAAA C C C C GAGAAGAAAAUGAAA	50′ tataa**ctcttcttttacttt**aacta 3′	5′UTR and 36–50
TFO2	AAAAGGAAAA C C C C AAAAGGAAAA	5′ gaaaa**ttttcctttt**gatag 3′	3′UTR and 29335–29344
TFO3	GGAUAAGAGGAA C C C C GGACAAGAGGAA	5′ ttaaa**cctgttctcctt**accga 3′	ORF1a/1b and 530–541
TFO4	AAAGAGGGGAGAA C C C C AAAGAGGUGAGAA	5′ cagga**tttctccactctt**agttc 3′	ORF1a/1b and 7399–7411
TFO5	AAAGGGAAGAAAGA C C C C AAAGUGAAGAAAGA	5′ aggag**tttcacttctttct**accat 3′	ORF1a/1b and 14048–14061
TFO7**	UUUUUAUUUUUAU C C C C UUUUUAUUUUUAU	—	—

*Highlighted in bold indicated the binding region.

**Unrelated circular TFO.

**Table 2:** Primers sequences used in the amplification of FIPV and H1NI viruses.

Target	Primer	Primer sequence (5′ to 3′)	Location	Reference	Amplicon size (bp)
FIPV	FIPV-F	GGCAACCCGATGTTTAAAACTGG	29082-29105	Herrewegh et al. (1995) [20]	223
	FIPV-R	CACTAGATCCAGACGTTAGCTC	29305-29283		
H1N1	M2-F	GGC AAA TGG TAC AGG CAA TG	636-655	Mehrbod et al. (2012) [21]	147
	M2-R	AGC AAC GAG AGG ATC ACT TG	760-779		

**Table 3:** *K*_*a*_ and *K*_*d*_ values related to circular TFO RNAs.

Circular TFO RNA	*K*_*a*_ (association constant)	*K*_d_* (dissociation constant) (nM)
1	28708286	34.8
2	—	—
3	9359929.6	107
4	8446828	118
5	1100864.4	908

*K_*d*_ is in reverse relation with *K*_*a*_.
